# Exploring the Antioxidant Potential of *Tragia volubilis* L.: Mitigating Chemotherapeutic Effects of Doxorubicin on Tumor Cells

**DOI:** 10.3390/antiox12112003

**Published:** 2023-11-14

**Authors:** Natalia Bailon-Moscoso, José Coronel-Hidalgo, Rodrigo Duarte-Casar, Luis Miguel Guamán-Ortiz, Jorge G. Figueroa, Juan Carlos Romero-Benavides

**Affiliations:** 1 Departamento de Ciencias de la Salud, Facultad de Ciencias de la Salud, Universidad Técnica Particular de Loja, Loja 1101608, Ecuador; jicoronel2@utpl.edu.ec (J.C.-H.); lmguaman@utpl.edu.ec (L.M.G.-O.); 2 Carrera de Bioquímica y Farmacia, Facultad de Ciencias Exactas y Naturales, Universidad Técnica Particular de Loja, Loja 1101608, Ecuador; 3 Maestría en Química Aplicada, Facultad de Ciencias Exactas y Naturales, Universidad Técnica Particular de Loja, Loja 1101608, Ecuador; rduarte@utpl.edu.ec; 4 Tecnología Superior en Gestión Culinaria, Pontificia Universidad Católica del Ecuador—Sede Manabí, Portoviejo 130103, Ecuador; 5 Departamento de Química, Facultad de Ciencias Exactas y Naturales, Universidad Técnica Particular de Loja, Loja 1101608, Ecuador; jgfigueroa@utpl.edu.ec (J.G.F.); jcromerob@utpl.edu.ec (J.C.R.-B.)

**Keywords:** *Tragia*, antioxidant activity, Doxorubicin, cytotoxicity, antigenotoxic

## Abstract

Several plants of the genus *Tragia* L. have shown antibacterial, fungicidal, and antiproliferative activity, among other types of activities; however, most species of the genus have not been investigated. *Tragia volubilis* L. is native to tropical America and Africa, and although it has been reported as medicinal in the literature, it has not been thoroughly investigated. In this study, the phytochemical screening, isolation, and identification of compounds and the determination of the antioxidant activity of the aqueous extract of *Tragia volubilis* L. and its partitions were carried out. Ethyl acetate and *n*-butanol partitions of the extract present high antioxidant activity according to the Antioxidant Activity Index. Due to their activity, these partitions were tested on RKO cells as a representative model, both individually and in combination with Doxorubicin. It was found that the partitions significantly reduced the effect of Doxorubicin, as well as the expression of proteins involved in DNA damage and cell death. While the reduction of the chemotherapeutic effect of Doxorubicin on tumor cells may not be a desired outcome in therapeutic settings, the findings of the study are valuable in revealing the antioxidant potential of *Tragia volubilis* L. and its partitions. This highlights the importance of carefully regulating the application of antioxidants, especially in the context of cancer chemotherapy.

## 1. Introduction

In recent years, antioxidants, due to their ability to protect cells from oxidative damage, have emerged as promising preventive and/or therapeutic agents for acute or chronic diseases caused by oxidative stress, such as cancer [1,2]. However, despite the evidence of the benefits that antioxidants promote in human health, their use presents much inconsistencies in the literature [3]. In cancer, where the underlying cause is malignant changes in cells primarily caused by oxidative damage [4], their use has been suggested to interfere with the metabolic activation of chemical carcinogens and to either promote the repair of premalignant lesions or inhibit their development [3]. However, their use is controversial, as randomized clinical trials have shown that antioxidant supplementation may increase the incidence and mortality of various types of cancer [5,6,7,8]. Furthermore, it has been suggested that the concomitant use of antioxidants with chemotherapy may have negative effects on treatment by interfering with the effectiveness of antineoplastic agents that rely on generating oxidative stress as their mechanism of action [9]. Currently, there is no definitive consensus on the use of antioxidants in cancer therapy.

Plants have been an important source of exogenous antioxidants over time, with an estimated two-thirds of the world’s plant species having medicinal significance and excellent antioxidant capacity [10]. Phenolic compounds and flavonoids are the most common secondary metabolites with antioxidant activity in plants [11,12,13]. They perform functions such as scavenging free radicals, donating hydrogen atoms or electrons, or chelating metal cations [14]. In recent years, there has been a growing interest in the research of these natural antioxidants due to their potential to counteract the harmful effects of oxidative damage induced by reactive oxygen species (ROS) [15].

*Euphorbiaceae* is a plant family with many and potent medicinal species [16]. Within this family, the *Tragia* genus is traditional in Africa and Asia for a variety of ailments [17], with its New World species lagging behind in the study of their medicinal properties. The main activity of *Tragia* extracts and essential oils is antibacterial, antimycotic, and antiproliferative. Around one-sixth of the 154 species in the genus are reported as medicinal, and the bulk of the research centers on four of these species, namely *Tragia involucrata* Linn., *Tragia spathulata* Benth., *Tragia benthamii* Baker, and *Tragia plukenetii* Radcl. The medicinal use of several more species that have not been well-studied is also documented in the literature [18,19].

A New World *Tragia* species that has ample geographical distribution and serves as the lectotype for the genus is *Tragia volubilis* L.; it is present not only in inter-tropical America but also in Africa, where it is considered an introduced species. It is considered nonspecifically medicinal [20] and is reported as being a diuretic [21] and can be used for rheumatism and wound healing [22]. The methanolic extract of its aerial parts exhibits high antioxidant activity, and four flavonoids, namely avicularin, quercitrin, afzelin, and amentoflavone, were isolated from it [23]. The objective of this study is to characterize the aqueous extract of the aerial parts of *T. volubilis*, evaluate its antioxidant activity, and examine the effect in the cytotoxicity and capacity of the resulting fractions of the extract against Doxorubicin, one of the most widely used and effective antineoplastic agents for the treatment of leukemia, lymphoma, and various types of solid tumors [24].

## 2. Materials and Methods

### 2.1. Plant Material

**Figure 1:**
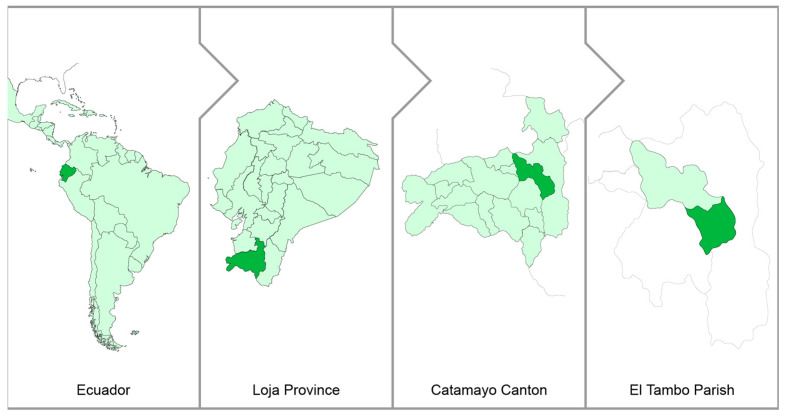
Collection area of *T. volubilis* L. used in this work. Areas in dark green correspond to the geographical division stated in the panel.

The collection of plant material for this study was completed in El Tambo parish, Catamayo canton, in the Loja province in Southern Ecuador (Figure 1). A total of 1987 g of the aerial parts of *Tragia volubilis* L. was collected. The species was identified by Fani Tinitana, PhD, and a voucher specimen number HUTPL7853 was deposited at the Herbarium of Universidad Técnica Particular de Loja, Ecuador. The specimen was collected under the Ecuadorian Ministry of Environment, Water, and Ecological Transition registry number MAE-DNB-CM-2016-0048, dated 20 September 2016.

The collected plant material was held under airflow for seven days at 30 °C until dry.

### 2.2. Extract and Partitions of Tragia volubilis

Once dried, the plant material was extracted with water at room temperature for 72 h (static maceration). The resulting liquid was lyophilized in a Labconco model 7754047 freeze drier (Kansas City, MO, USA) to yield the aqueous extract. The extract was sequentially fractioned with solvents of increasing polarity: hexane, dichloromethane, ethyl acetate, *n*-butanol (Merck KGaA, Darmstadt, Germany), and water. Then, 50.1 g of aqueous extract was dissolved in 1.5 L distilled water and extracted three times with 1500 mL of the solvent, concentrating the solvent partition by rotary evaporation and reusing the recovered solvent. The partition was concentrated to dryness and stored at −18 °C. In the case of the aqueous residue, concentration was performed through freeze drying.

### 2.3. Phytochemical Screening

Phytochemical screening of the extracts was performed to identify compound families present in the extract partitions, according to the methodology shown in Mandal et al. [25], using the assays detailed in Silva-Rivas et al. [26]. The screening tested for the presence or absence of proteins, carbohydrates, fats, alkaloids, terpenoids, flavonoids, saponins, quinones, and tannins.

### 2.4. Isolation of Secondary Metabolites

The ethyl acetate partition of the aqueous extract of *T. volubilis* was fractioned using flash chromatography (Buchi Reveleris^®^ PREP, Flawil, Switzerland). Direct phase separations were performed with 12 g direct silica columns and hexane–ethyl acetate–methanol elution gradients.

### 2.5. Characterization and Identification

Once isolated, a secondary metabolite was identified through NMR spectra. The ^1^H- and ^13^C spectra were obtained on a BRUKER Ascend 500 MHz spectrometer (Billerica, MA, USA), at 298 K, using deuterated methanol as the solvent. The tentative interpretation of the spectra was compared with published results for confirmation.

### 2.6. Antioxidant Capacity

The total phenolic content (TPC) of the partitions of the extract was measured using the Folin–Ciocâlteu method [27]. Samples of the extract were diluted in wells of a 96-well plate. To 10 µL of those, 50 µL of Folin–Ciocâlteu reagent (Sigma-Aldrich, St. Louis, MO, USA) was added, followed by 10 min of homogenization. Then, 150 µL of 7.5% *w*/*w* Na_2_CO_3_ (Sigma-Aldrich, St. Louis, MO, USA) solution was added, and distilled water was added to complete 1 mL. Afterward, the wells were homogenized again for 5 min. Absorbance was read at 760 nm on a Bio Tek Epoch 2 microplate reader (BioTek Instruments Inc., Winooski, VT, USA), and the values compared to a linear gallic acid calibration curve. TPC is expressed in gallic acid equivalents (GAEs) per gram of extract.

The antioxidant capacity of the extract and the partitions was measured through three assays: ABTS (2,2′-azino-bis (3-ethylbenzothiazoline-6-sulfonic acid)), FRAP (ferric reducing antioxidant power), and DPPH (2,2-diphenyl-1-picrylhydrazyl), all presented as Trolox (6-hydroxy-2,5,7,8-tetramethylchroman-2-carboxylic acid) equivalents. All results are expressed as the average ± standard deviation of three repetitions. The Antioxidant Activity Index (AAI) is a value that is independent of the concentrations of both the sample and DPPH [28], and it was calculated as the quotient between the final DPPH concentration and the IC_50_ for each sample in order to better ascertain the antioxidant activity.

### 2.7. Cell Culture

Human colorectal carcinoma RKO (wt p53) (ATCC^®^ CRL-2577™). Cells were grown at 37 °C and in a 5% CO_2_ atmosphere, in DMEM medium (GIBCO, Grand Island, NY, USA) supplemented with FBS 10% (Sigma-Aldrich, St. Louis, MO, USA), penicillin 0.1 mg/mL, streptomycin 100 U/mL, and glutamine 2 mM (GIBCO, Grand Island, NY, USA).

### 2.8. Viability Assay

Cell viability was analyzed using the MTS metabolic viability assay, which measures the mitochondrial activity of live cells. In summary, cells were seeded in 96-well culture plates at a density of 3 × 10^3^ cells in 100 µL per well. After 24 h of incubation, the medium was changed, and different treatments were added, followed by an additional 48-h incubation period. RKO cells were treated with individual partitions of ethyl acetate and *n*-butanol of the aqueous extract at a concentration of 100 µg/mL, as well as in combination with 0.05 µM Doxorubicin (Dxo, Sigma-Aldrich, St. Louis, MO, USA). The blank (supplemented medium), negative control, and positive control using 0.05 µM Dxo were included. Four hours prior to completing the treatment, 20 µL of CellTiter 96^®^ AQueous One Soln. Cell Prol. reagent (Promega, Madison, WI, USA) was added to each well. The absorbance of each sample was measured using a spectrophotometer (Epoch 2, BioTek, Santa Clara, CA, USA) at a wavelength of 490 nm. The experiments were performed in triplicate. Data obtained from untreated cells were used as reference values (100% viability) to normalize the absorbance of the treated samples [29].

### 2.9. Morphological Analysis

To determine the effect induced by individual and combined partitions of ethyl acetate and *n*-butanol with Dxo on the RKO cell line, 2 × 10^5^ cells were seeded in a 6-well plate. After 24 h of incubation, the treatments mentioned above for the viability assay were applied. After 48 h of cell treatment, they were observed and photographed under the Axio Observer 7 microscope (ZEISS, White Plains, NY, USA) at 400× magnification.

### 2.10. Western Blot

For the Western blot assay, 1 × 10^6^ RKO cells were seeded in T75 flasks. The cells were then treated individually with partitions of ethyl acetate and *n*-butanol at a concentration of 100 µg/mL, both alone and in combination with 0.05 µM Dxo. The methodology was applied as described by Bailon-Moscoso et al. [30]. In summary, 30 or 50 μg of total protein was separated by SDS-PAGE on a 7–15% gel and subsequently transferred to a polyvinylidene difluoride (PVDF) membrane. After blocking the membrane with 5% (*w*/*v*) skimmed milk, the target proteins were immunodetected using specific antibodies: from Cell Signaling Technology (H2AX: #7631; γH2AX: #9718; Phospho-p53 (Ser15): #9284, Danvers, MA, USA) and Santa Cruz Biotechnology (p53: sc-126; p21: sc-817; PARP1: sc-53643; Actin: sc-58673) at the manufacturer-recommended dilution. Following a series of washes, corresponding secondary antibodies from Santa Cruz Biotechnology (goat anti-rabbit IgG-HRP: sc-2054; m-IgGκ BP-HRP: sc-516102, Dallas, TX, USA) were applied at a 1:5000 dilution. Immunoreactive bands were visualized using an enhanced chemiluminescence kit (Millipore/Sigma-Aldrich, St. Louis, MO, USA).

## 3. Results

### 3.1. Phytochemical Screening and Compound

**Figure 2:**
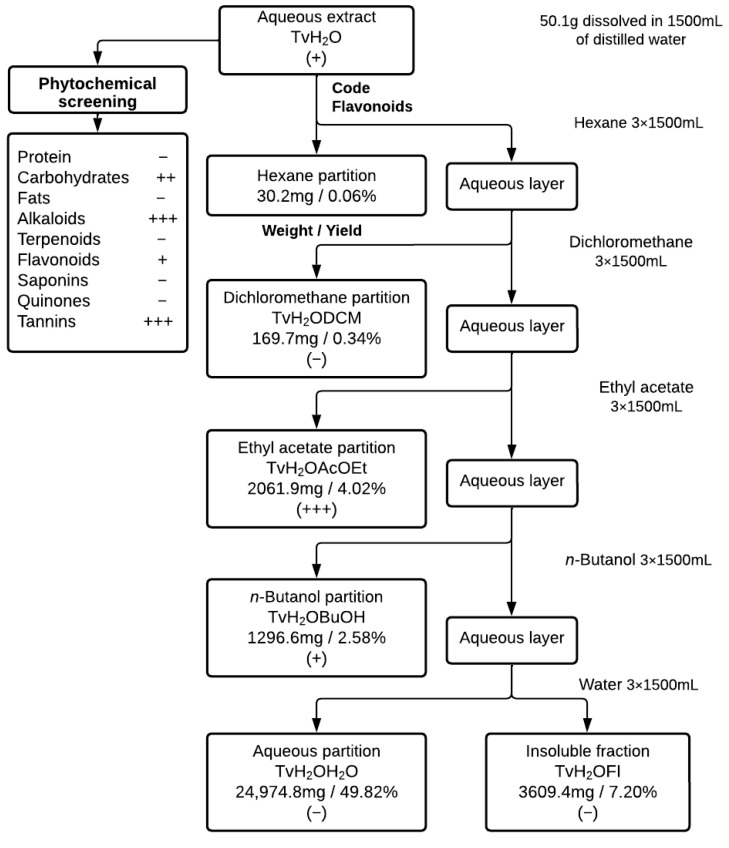
Partitions, weight, yield, and flavonoid presence. Key: −, negative; +, scant presence; ++, appreciable presence; +++, abundant presence.

The working extract and partitions, as well as the compound families contained, are detailed in Figure 2. The screening shows the presence of compound classes that correspond to those of other species in the genus, such as *Tragia involucrata* L. [31], *Tragia pungens* (Forssk.) Müll.Arg. [32], and *Tragia benthamii* Baker [33], and similar to the methanolic extract of the same species [23], with differences such as lesser flavonoid presence and terpenoid absence in the aqueous extract attributable to the increased solvent polarity. Differences with other species, such as the absence of flavonoids in *T. benthamii*, can be attributed partially to the extraction solvent and procedure followed. Figure 2 shows the sequence of this work.

**Figure 3:**
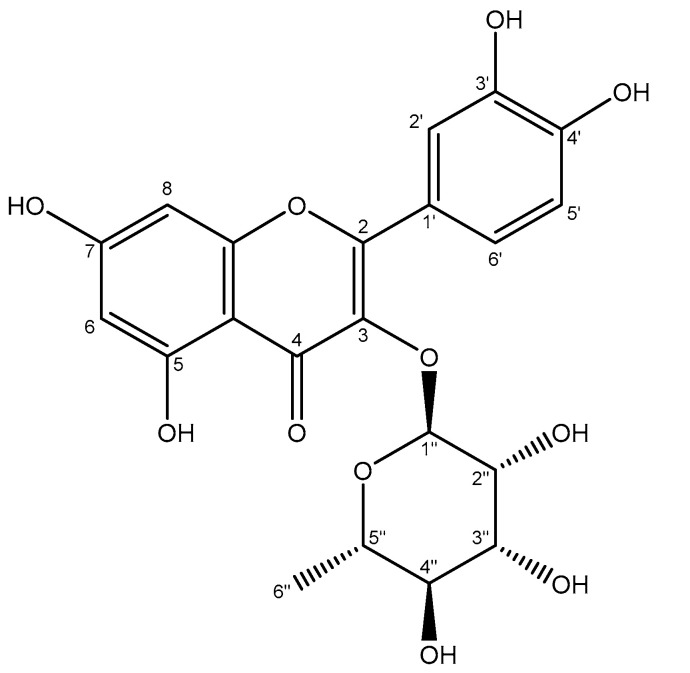
Structure of quercitrin, with carbon atoms numbered.

Quercitrin (Figure 3) was isolated and identified from the ethyl acetate partition of the aqueous extract—chosen for its highest antioxidant activity among the partitions. The chemical characterization of quercitrin was performed based on the following signals of ^1^H–NMR (500 MHz, CD_3_OD, δ ppm, J/Hz): 7.33 (1H, d, 1.8. H-2′), 7.30 (1H, dd, 8.35 1.9, H-6′), 6.90 (1H, d, 8.35, H-5′), 6.35 (1H, brs, H-8), 6.19 (1H, d, 1.5, H-6), 5.30 (1H d 1.1 H-1″), 4.19–4.23 (1H, m, H-2″), 3.74 (1H, dd, 9.5 3.3, H-3″), 3.39–3.45 (1H, m, H-5″), 3.33–3.35 (1H, m, H-4″), and 0.93 (3H, d, 6.2, H-6″). ^13^C NMR (CD_3_OD, δ ppm): 70.5 (2″), 70.6 (3″), 70.7 (5″), 71.8 (4″), 93.4 (8), 98.5 (6), 104.5 (10), 104.5 (1″), 115.0 (5′), 115.5 (2′), 121.5 (6′), 121.6 (1′), 134.8 (3), 145.0 (3′), 148.5 (4′), 157.1 (9), 157.9 (2), 161.7 (5), 164.5 (7), and 178.3 (4). These results were and confirmed via a comparison with published results [34].

### 3.2. Antioxidant Activity

The antioxidant activity evaluations were conducted on various partitions of the aqueous extract of *T. volubilis*. Several parameters were measured (Table 1). Among them, the IC_50_ and AAI values were determined, representing the amount of extract required to neutralize 50% of the DPPH radical and an Antioxidant Activity Index independent from the sample and DPPH concentrations.

**Figure 4:**
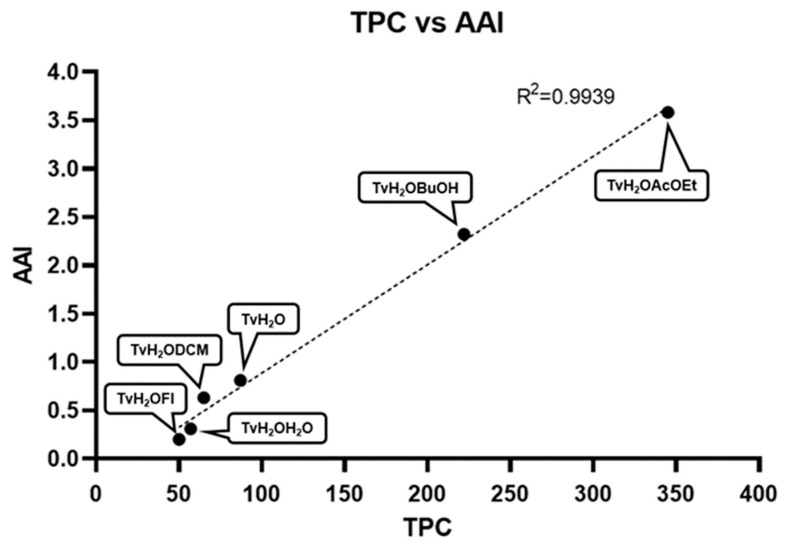
Correlation between total phenolic content and AAI.

Both the aqueous/ethyl acetate (TvH_2_OAcOEt) and aqueous/*n*-butanol (TvH_2_OBuOH) partitions exhibit very strong antioxidant activity (AAI > 2), greater than that of the methanolic extract of the species (AAI = 1.14) [23], while both aqueous and aqueous/DCM extract show moderate activity (AAI > 0.5) [35]. There is a strong linear relationship between TPC and AAI (R^2^ = 0.9939) that supports the assumption that most of the antioxidant capacity of the extracts is attributable to phenolic compounds (Figure 4).

### 3.3. The Partitions of Ethyl Acetate and n-Butanol from Aqueous Extract of T. volubilis Protect RKO Cells from Dxo Cytotoxicity

**Figure 5:**
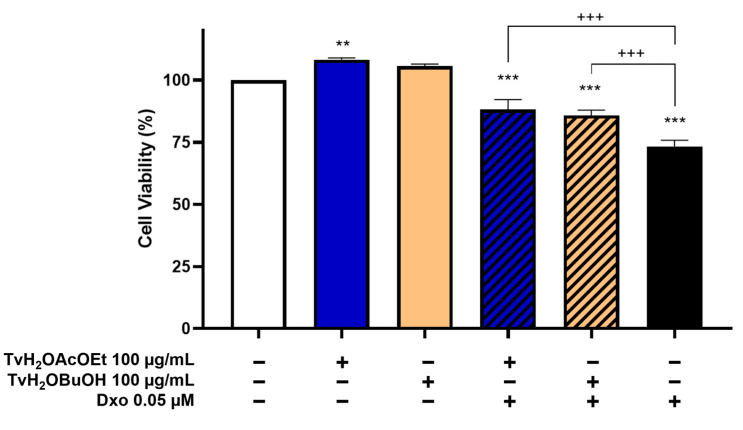
Viability of RKO cell line after 48-h treatment with 100 µg/mL of *T. volubilis* partitions alone and in combination with Dxo. Cell viability is expressed as a percentage relative to the control (described as 100%). (−) Absence, (+) Presence. Data represent mean ± SD of three independent experiments. Statistical analysis was performed using one-way ANOVA, followed by Tukey’s test. ** *p* < 0.01, *** *p* < 0.001 vs. control; +++ *p* < 0.001 vs. Dxo.

The partitions with the highest antioxidant activity, namely ethyl acetate (TvH_2_OAcOEt) and *n*-butanol (TvH_2_OBuOH), both individually and in combination with Dxo, demonstrated a significant cytoprotective effect on the viability of RKO cells during the evaluation of growth and viability effects (Figure 5). The cytotoxicity of Dxo was reduced by 12% and 15% with the concomitant administration of TvH_2_OBuOH and TvH_2_OAcOEt, respectively.

**Figure 6:**
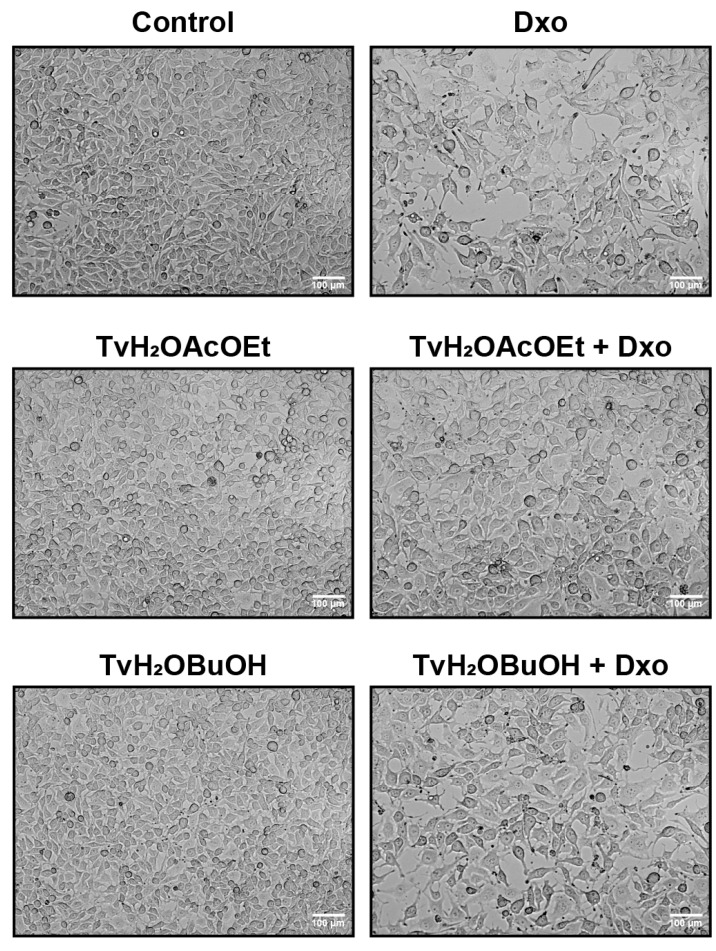
Morphology of RKO cells after 48-h treatment with 100 µg/mL of *T. volubilis* partitions alone and in combination with Dxo 0.05 µM.

Similarly, the morphological analysis showed the cytoprotective effect of TvH_2_OBuOH and TvH_2_OAcOEt on the viability of RKO cells treated with Dxo, with a higher cell confluence observed in the treatments with the extracts combined with Dxo compared to cells treated with Dxo alone (Figure 6).

### 3.4. The Partitions of Ethyl Acetate and n-Butanol from Aqueous Extract of T. volubilis Reduce Genotoxic Damage and Cell Death Induced by Dxo in RKO Cells

**Figure 7:**
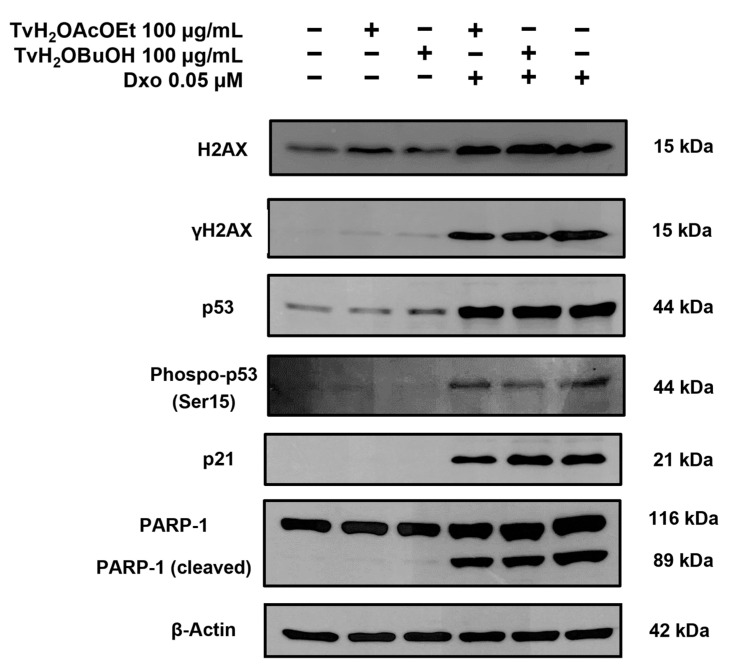
Proteins involved in genotoxic damage and cell death. RKO cells were treated for 48 h with *T. volubilis* partitions alone and in combination with Dxo. (−) Absence, (+) Presence. Subsequently, protein expression levels were examined using Western blot in the RKO cell line. The relative protein expression was quantified using densitometry, with β-actin used as a control.

**Figure 8:**
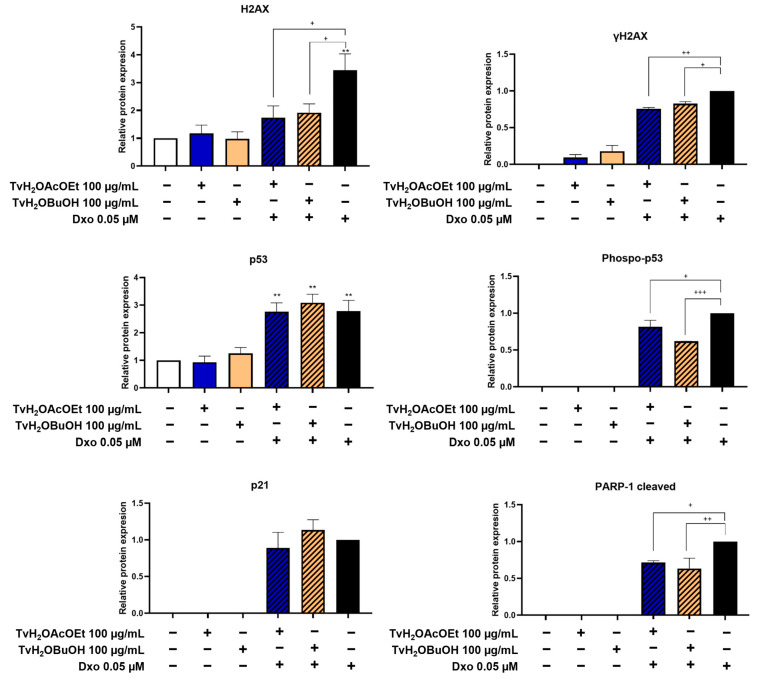
Quantitative analysis of protein expression levels involved in genotoxic damage and cell death. Data represent the mean ± SD of two independent experiments. (−) Absence, (+) Presence Statistical analysis was performed using one-way ANOVA, followed by Tukey’s test. ** *p* < 0.01 vs. control; + *p* < 0.05, ++ *p* < 0.01, +++ *p* < 0.001 vs. Dxo.

The levels of expression of proteins related to genotoxic damage and cell death were quantified using the Western blot assay in RKO cell line cells. To determine DNA damage, the levels of the DNA damage biomarker γH2AX were evaluated in the treatments of TvH_2_OBuOH and TvH_2_OAcOEt alone and in combination with Dxo. It was found that the treatments with the partitions in combination with Dxo showed the capacity to significantly decrease the expression of H2AX and its phosphorylated form γH2AX compared to the Dxo treatment, indicating their potential to attenuate genotoxic damage (Figure 7 and Figure 8).

The expressions of proteins controlling cell cycle progression and involved in the damage of DNA and apoptosis were assessed. The quantitative results of the expression levels of p53 and p21 in the combined treatments did not show significant changes. However, upon further examination, a lower expression of the phosphorylated form of p53 (Phospo-p53 (Ser15)) was observed in the combined treatments, with statistically significant results compared to the Dxo treatment. The results of the expression of PARP-1 (apoptosis marker) demonstrated a significant decrease in its cleavage in the combined treatments of TvH_2_OBuOH and TvH_2_OAcOEt with Dxo compared to the treatment with Dxo.

## 4. Discussion

Currently, antioxidants have emerged as preventive or therapeutic agents for diseases caused by oxidative stress, due to their ability to protect macromolecules from oxidative damage. Natural antioxidants derived from plants, such as phenolic compounds, have gained increasing interest for their anticancer properties [36,37,38]. However, controversy has arisen in recent years regarding the use of antioxidants, with suggestions that administering antioxidants during cancer chemotherapy could reduce treatment efficacy. This is because certain chemotherapeutic agents rely on the production of free radicals and ROS as part of their mechanism of action [39], and antioxidants may neutralize these radicals and negatively interfere with treatment by protecting cancer cells during therapy or inducing the proliferation of residual cancer cells [40]. Currently, there is no definitive consensus on the use of antioxidants in cancer therapy. Different clinical trials have shown beneficial effects [41,42,43,44]; however, in contrast, other trials [45,46,47,48,49] have indicated a trend toward worse survival in patients treated with antioxidants while receiving chemotherapy.

In the present study, the results showed that the ethyl acetate (TvH_2_OAcOEt) and *n*-butanol (TvH_2_OBuOH) fractions obtained from the aqueous extracts of the aerial parts of *T. volubilis* have high antioxidant activity and a good correlation with their total phenolic content. Phytochemical screening tests indicated a low and abundant presence of flavonoids in TvH_2_OBuOH and TvH_2_OAcOEt, respectively, and the glycosylated flavonoid quercitrin was isolated and identified in TvH_2_OAcOEt. Other species of the genus *Tragia* have also been reported to have potent antioxidant activity [50] and have also been attributed to phenolic-type compounds [51].

Subsequently, the effects of TvH_2_OBuOH and TvH_2_OAcOEt as natural antioxidants in combination with Doxorubicin were investigated in RKO cells. The results of the viability assays indicated the potential of the extracts to significantly reduce the cytotoxicity of Doxorubicin, an anthracycline antibiotic that exerts its mechanism of action on cancer cells by disrupting DNA repair mediated by topoisomerase II and generating free radicals [52]. Dxo can generate free radicals through two mechanisms: The first is an enzymatic mechanism where Dxo is reduced to its semiquinone by oxidases such as nicotinamide adenine dinucleotide phosphate (NADPH), and this semiquinone can autoxidize in the presence of oxygen, producing superoxide radicals [53]. The second mechanism involves the reaction of Dxo with iron (Fe^3+^), and this complex can reduce oxygen to hydrogen peroxide (H_2_O_2_) and other free radicals [54]. Consequently, the increase in ROS and free radicals in cells contributes to nuclear and mitochondrial DNA damage, simultaneously triggering lipid peroxidation and ultimately inducing cell death [55]. Therefore, antioxidants, due to their ability to protect cells from oxidative damage, could partially inhibit the cytotoxic activity of this drug [56,57]. Several studies have shown a protective effect of quercitrin on normal cells. In this regard, Li et al. [58] described how treatment with quercitrin (10–100 µg/mL) protected mesenchymal stem cells from oxidative damage by indirectly (Fe^2+^ chelation) or directly eliminating ROS. Ham et al. [59] reported a reduction in intracellular ROS, the inhibition of lipid peroxidation, and apoptosis due to oxidative stress after V79-4 lung cells received pretreatment with quercitrin. Other studies have demonstrated that quercitrin, due to the plurality of hydroxyl (OH) groups in its structure, has the ability to eliminate free radicals, sequester metal ions, and form metal ion chelates [60,61]. In addition, quercetin attenuated the cytotoxic effect of Dxo in H9C2 cardiomyocyte cells, suggesting that quercetin could eliminate ROS and reduce oxidative damage [62]. Similarly, numerous in vitro studies have indicated that various types of flavonoids, due to their antioxidant properties, have cytoprotective effects by reducing cell damage induced by oxidative stress [63,64,65,66,67] and attenuating the cytotoxicity induced by Dxo [68,69,70]. Based on the above considerations, our results indicate that *T. volubilis* extracts, due to their high phenolic content and antioxidant activity, exert a cytoprotective effect by reducing oxidative stress and cell damage induced by Dxo, thereby decreasing the susceptibility of RKO cells to cell death and, consequently, increasing the survival and viability of tumor cells.

The cytoprotective effect of TvH_2_OBuOH and TvH_2_OAcOEt may be largely related to the reduction of genotoxic damage, as shown in Western blot assays. The oxidative stress produced by Dxo induces single-strand breaks (SSBs) and double-strand breaks (DSBs) [55,71]. In response to DNA damage, the ataxia telangiectasia mutated (ATM) and ATM and Rad3-related (ATR) kinases cause the variant histone H2A (H2AX) to rapidly phosphorylate at serine 139 to form γH2AX. γH2AX is considered a sensitive indicator of genotoxic damage [72]. Our results showed the ability of the extracts to significantly decrease the expression of H2AX and its phosphorylated form, γH2AX, compared to the treatment with Dxo. Consistent with our findings, in vitro studies have reported increased γH2AX expression in response to Dxo treatment and oxidative stress [73,74]. Similarly, numerous in vitro assays have demonstrated the reduction of DNA damage by various agents and stimuli through the antioxidant activity of phenolic compounds [75,76,77,78,79,80]. Additionally, they have shown a decrease in γH2AX formation [81,82,83].

Although no changes were observed in the expression of p53 and p21 proteins, the phosphorylated form of p53 was observed in the combined treatments. When DNA damage occurs, rapid and substantial phosphorylation occurs at multiple sites of p53, initially through the phosphorylation of serine 15 by ATM or ATR, which is activated in parallel with H2AX phosphorylation. The activation of p53 induces its transcriptional and pro-apoptotic function [84]. Regarding our results, Ju et al. [85] observed that the induction and phosphorylation of p53 (Phospho-p53 (Ser15)) in response to Dxo in RKO cells are mainly controlled at the post-translational level. On the other hand, a study on the HCT-116 cell line suggested that the expression of p21 in response to various stimuli that induce DNA damage seems to be independent of the increase in p53 phosphorylation [86].

The latest observations showed a significant decrease in PARP-1 cleaved in TvH_2_OBuOH and TvH_2_OAcOEt treatments combined with Dxo compared to Dxo treatment. These results reinforce the idea that the extracts, due to their antioxidant capacity, inhibit Dxo-induced cell death by reducing its oxidative damage. According to the literature, the normal function of PARP-1 is the routine repair of DNA damage by adding poly(ADP-ribose) polymers in response to various cellular stresses; however, during apoptosis, PARP-1 is cleaved into 89 and 24 kDa fragments by executioner caspases 3 and 7, becoming cleaved PARP-1, a hallmark of apoptosis [87]. In line with our results, Dong et al. [88] reported that pretreatment with the flavonoid quercetin decreased the levels of cleaved PARP-1 and reduced the percentage of apoptosis induced by Dxo (5 µM) in H9C2 cells by reducing oxidative stress [88]. Therefore, based on our findings, we suggest that the reduction in cleaved PARP-1 expression is related to the decrease in genotoxic damage, as confirmed by γH2AX expression and lower p53 phosphorylation, leading to lower levels of pro-apoptotic proteins and resulting in reduced levels of cleaved PARP-1 and a decrease in the percentage of cell death.

## 5. Conclusions

Overall, our results suggest that the TvH_2_OBuOH and TvH_2_OAcOEt extracts from *T. volubilis*, due to their high antioxidant capacity and phenolic content, provide a significant viability effect to RKO cells by attenuating the oxidative damage caused by Dxo. This effect leads to a reduction in genotoxic damage and lower p53 phosphorylation, resulting in lower levels of pro-apoptotic proteins and, consequently, increasing the survival and viability of tumor cells. However, it is important to validate these results through additional assays and different experimental models.

The potential interactions between antioxidants and chemotherapy agents, like Doxorubicin, need to be thoroughly understood to avoid any interference with the desired cytotoxic effects on cancer cells. The improper timing or dosage of antioxidants during chemotherapy may compromise the efficacy of cancer treatment by reducing the ability of drugs to target cancer cells effectively.

It is crucial to address the limitations of the study to properly contextualize the findings and provide a balanced view of the conducted research. In this study, we identified some limitations that deserve mentioning. Firstly, the lack of comparison of our results with other cell lines represents a significant limitation, as it restricts the generalizability of our findings to a specific context. In future investigations, it is essential to include a variety of cell lines to evaluate the different extracts and partitions obtained from *Tragia volubilis* L., providing a more comprehensive understanding of its biological activity. Furthermore, although a reduction in the cytotoxic effect of Doxorubicin was observed in the presence of *Tragia volubilis* L. partitions, it is important to consider evaluating the activity of the extract on antioxidant enzymes and ROS/RNS. Additionally, it is crucial to assess the results in an in vivo environment, where a series of additional biological factors, such as bioavailability, toxicity, and stability, come into play. Also, from the chemical standpoint, compounds from the *n*-butanol partition could not be isolated using the procedure used in this study.

Therefore, future research should focus on elucidating the optimal conditions for the application of antioxidants during cancer therapy to ensure that their cytoprotective effects do not interfere with the therapeutic goals of chemotherapy. For a better understanding of the mechanisms and potential interactions between antioxidants and chemotherapy agents, more targeted and personalized treatment strategies could be developed to maximize the benefits of both approaches, while also minimizing potential drawbacks.

## Figures and Tables

**Table 1:** Antioxidant activity of *T. volubilis* extracts.

Extract/Partition	TPC mg GAE/g Extract	ABTS μmol TE/g Extract	FRAP μmol TE/g Extract	DPPH μmol TE/g Extract	IC_50_ mg Extract/mg DPPH	AAI [DPPH] (μg ml^−1^)/IC_50_
TvH_2_O	87 ± 2	1789 ± 8	1079 ± 24	417 ± 10	1.81 ± 0.06	0.81 ± 0.03
TvH_2_ODCM	65 ± 3	1494 ± 42	724 ± 17	271 ± 9	2.38 ± 0.14	0.63 ± 0.01
TvH_2_OAcOEt	345 ± 26	4421 ± 336	4315 ± 35	2107 ± 35	0.42 ± 0.02	3.58 ± 0.10
TvH_2_OBuOH	222 ± 2	4170 ± 63	3083 ± 47	1252 ± 77	0.64 ± 0.04	2.32 ± 0.07
TvH_2_OH_2_O	57 ± 0	1051 ± 5	631 ± 6	177 ± 1	4.83 ± 0.12	0.31 ± 0.01
TvH_2_OFI	50 ± 1	1005 ± 25	555 ± 10	170 ± 5	7.58 ± 0.62	0.20 ± 0.01

TPC = total phenolic content; GAE = gallic acid equivalent; TE = Trolox equivalent; ABTS = 2,2′-azino-bis (3-ethylbenzothiazoline-6-sulfonic acid) method; FRAP = ferric reducing antioxidant power; DPPH = 2,2-diphenyl-1-picrylhydrazyl method; IC_50_ = half maximal inhibitory concentration; AAI = Antioxidant Activity Index.

## Data Availability

Data is contained within the article.
